# Data-driven analysis of kappa opioid receptor binding in major depressive disorder measured by positron emission tomography

**DOI:** 10.1038/s41398-021-01729-5

**Published:** 2021-11-27

**Authors:** Kelly Smart, Ashley Yttredahl, Maria A. Oquendo, J. John Mann, Ansel T. Hillmer, Richard E. Carson, Jeffrey M. Miller

**Affiliations:** 1 Yale PET Center, Yale School of Medicine, New Haven, CT USA; 2 Molecular Imaging and Neuropathology Area, New York State Psychiatric Institute, New York, NY USA; 3 Department of Psychiatry, Columbia University, New York, NY USA; 4 Department of Psychiatry, Perelman School of Medicine, University of Pennsylvania, PA, CT USA; 5 Department of Psychiatry, Yale School of Medicine, New Haven, CT USA

**Keywords:** Molecular neuroscience, Depression

## Abstract

Preclinical studies have implicated kappa opioid receptors (KORs) in stress responses and depression-related behaviors, but evidence from human studies is limited. Here we present results of a secondary analysis of data acquired using positron emission tomography (PET) with the KOR radiotracer [^11^C]GR103545 in 10 unmedicated, currently depressed individuals with major depressive disorder (MDD; 32.6 ± 6.5 years, 5 women) and 13 healthy volunteers (34.8 ± 10 years, 6 women). Independent component analysis was performed to identify spatial patterns of coherent variance in KOR binding (tracer volume of distribution, *V*_T_) across all subjects. Expression of each component was compared between groups and relationships to symptoms were explored using the 17-item Hamilton Depression Rating Scale (HDRS). Three components of variation in KOR availability across ROIs were identified, spatially characterized by [^11^C]GR103545 *V*_T_ in (1) bilateral frontal lobe; (2) occipital and parietal cortices, right hippocampus, and putamen; and (3) right anterior cingulate, right superior frontal gyrus and insula, coupled to negative loading in left middle cingulate. In MDD patients, component 3 was negatively associated with symptom severity on the HDRS (*r* = −0.85, *p* = 0.0021). There were no group-wise differences in expression of any component between patients and controls. These preliminary findings suggest that KOR signaling in cortical regions relevant to depression, particularly right anterior cingulate, could reflect MDD pathophysiology.

## Introduction

Much preclinical literature implicates the kappa opioid system in maladaptive responses to stress and depression-like behaviors. The endogenous opioid dynorphin is released in response to stress, resulting in preferential activation of the kappa opioid receptor (KOR) [1]. Dynorphins are also released in response to activation of the hypothalamic-pituitary-adrenal axis in animals by administration of corticotropin releasing hormone [2]. In animal models, KOR agonists induce depression-like behaviors [3, 4], and KOR antagonists exert antidepressant-like effects [4–8]. KOR activation modulates several monoamine neurotransmitter systems. Of note, KOR activation inhibits dopaminergic output from the ventral tegmental area to the nucleus accumbens [3]. This has led to a focus on the role of KOR activation in reward processing and in anhedonia. In humans, kappa agonist medications elicit acute dysphoric and psychotogenic reactions [9, 10]. Several medications with KOR modulating effects including JNJ-67953964 and buprenorphine/samidorphan have been studied as antidepressant or anti-anhedonia treatments in humans [11–13].

Surprisingly, the role of the kappa opioid system in depression in vivo in humans is understudied. We previously compared regional brain KOR binding between individuals with current major depressive disorder (MDD) and healthy volunteers using positron emission tomography (PET) with the KOR-specific radiotracer [^11^C]GR103545. In that study, we examined a small number of a priori regions of interest (ROIs) based on animal findings: hippocampus, raphe nuclei, amygdala, and ventral striatum [14]. We did not observe a group difference in binding across these a priori ROIs. While a whole-brain voxel-wise exploratory analysis was conducted in that paper, statistical power was limited.

We now report the results of exploratory secondary analyses of these data using data-driven, whole-brain comparisons to determine whether there are alterations in KOR binding that were not captured in the hypothesis-driven previous work. To this end, we used independent component analysis (ICA) of PET data to identify brain regions with correlated patterns of KOR binding. This approach has three advantages: 1) it allows us to identify putative inherent structures of KOR maps; 2) it reduces data dimensionality dramatically, increasing statistical power, and 3) it is blind to subject group membership. These methods allowed us to identify possible network patterns of KOR/dynorphin signaling relevant to MDD.

## Materials and methods

Data from a previous study were re-analyzed [14]. Briefly, 10 participants with MDD and 13 age- and sex-matched healthy control volunteers (HCs) underwent medical and psychiatric screening. Diagnosis was established using the Structured Clinical Interview for the DSM-IV (SCID) [15] and severity of depressive symptoms was assessed using the 17-item Hamilton Depression Rating Scale (HDRS) [16] and the Beck Depression Inventory (BDI) [17]. MDD patients had a score on the HDRS ≥ 16 at the time of consent, were experiencing a current depressive episode, and were not taking antidepressant medications at the time of scan. The 17-item HDRS was administered by skilled raters with inter-rater reliability values (ICC) of 0.96. Participants had no physical or neurological illnesses and no history of alcohol or substance use disorders or of psychotic disorders. Each participant underwent a PET scan with [^11^C]GR103545 and a structural magnetic resonance imaging (MRI) scan for image registration and ROI definition. All participants provided informed consent prior to completing any study procedures. Study procedures were approved by the Institutional Review Boards of The New York State Psychiatric Institute and the Yale University School of Medicine.

### PET acquisition

[^11^C]GR103545, a selective kappa agonist radiotracer [18, 19], was synthesized and scans were performed as previously reported. Scans were acquired on a high resolution research tomograph (HRRT; Siemens/CTI). Following bolus injection of radiotracer (531 ± 332 MBq/nmol), dynamic PET data were acquired for 150 min. Blood samples were drawn from the subject’s radial artery for measurement of plasma radioactivity and fraction of unmetabolized parent compound at intervals throughout the scan to construct a metabolite-corrected arterial input function for kinetic analysis.

### PET outcome measure estimation

#### ROI-level analysis

Anatomical images were registered to the Montreal Neurological Institute 152 (MNI152) template using non-linear transformations with the Computational Anatomy Toolbox 12 (CAT12; http://www.neuro.uni-jena.de/cat/). Linear transformations were computed between motion-corrected PET data and anatomical MRI. Forty-nine ROIs (24 regions analyzed separately in left and right hemispheres plus midbrain Raphe nuclei) were defined using the Automated Anatomic Labeling atlas in template space and warped into subject PET space with the computed transformations. Time-activity curves were generated for each ROI. Tracer volume of distribution (*V*_T_), representing the sum of total specific binding, nonspecific binding, and free radiotracer in each ROI, was determined using multilinear analysis 1 (MA1) with the parameter t* set to 40 min [18]. To minimize the influence of extreme (nonphysiological) *V*_T_ estimates arising from noise in dynamic PET data, ROI values more than 3 SD above mean value were replaced with the median value for that ROI (total of 26 values out of 1127 total across all subjects and ROIs, i.e., 2.3%) based on conventional thresholds for identifying outliers.

#### Voxel-wise images

Whole-brain images of [^11^C]GR103545 *V*_T_ were generated using MA1. Dynamic data were processed with the HighlY constrained backPRojection (HYPR) method using a five-frame window [20] and smoothed with an 8 mm full width half maximum Gaussian filter to reduce noise in individual frames. Images were warped into template space using computed transforms. As in a previous analysis of voxel-level KOR PET data [21], *V*_T_ values below zero or above the 95^th^ percentile were replaced with the median of the 26 immediately adjacent voxels, and a template gray matter mask was applied prior to ICA.

### ICA

ICA is a data-driven method used to identify multiple sources of variance in a dataset, ‘unmixing’ observed signals into maximally independent components and differentiating relevant functional brain signals from variation related to noise [22]. In contrast to principal component analysis (PCA), which attempts to maximize variance accounted for in a dataset, the goal of ICA is to separate unknown sources in mixed data. ICA has been applied to PET data to perform target-specific quantification from PET data using a radiotracer with two distinct binding sites [23, 24] and to identify distinct sources of spatial variance in tracers with a single target, such as networks of synaptic density with [^11^C]UCB-J [25]. With [^11^C]GR103545, ICA offers the possibility of characterizing overlapping but distinct spatial distributions of KOR availability based on coherent patterns of variance across subjects. This may be a relevant approach for the KOR/dynorphin system given the local regulation of neuropeptide signaling and of dynorphin in particular. In contrast to neuromodulators such as dopamine and serotonin which are synthesized by well-characterized populations of projection neurons along specific circuits, neuropeptides like dynorphin are typically synthesized and released locally by many unrelated groups of neurons across various brain regions [26]. Their production, release, and downstream action at KOR is heavily regulated through overlapping, often regionally specific mechanisms [27, 28]. Therefore, ICA applied to [^11^C]GR103545 PET data may allowing unmixing of cross-regional patterns of KOR/dynorphin signaling with common regulatory influences or which are similarly implicated in or affected by MDD.

Spatial ICA was performed on ROI values as the primary analysis. In a further exploratory analysis, whole-brain images of [^11^C]GR103545 *V*_T_ were also analyzed using ICA to confirm that similar regional patterns would be captured and allow us to capture relevant variation within larger ROIs. For ROI and voxel-level analyses, regional values or images from all subjects were entered into ICA together. The global mean value was first subtracted from each subject’s data. The number of maximally independent components present in the voxel-level data was determined to be three using a minimum description length criterion approach validated for model order selection in neuroimaging data [29], and so three components were extracted in both analyses.

ICA estimates *M* statistically independent components of variation from data in *S* subjects, as well as an *M* x *S* mixing matrix. Here, ICA was applied to identify 3 components of coherent spatial variance in *V*_T_ values. Spatial pattern of each component is defined in a set of spatial intensity values at each ROI or voxel (*y*_m_, where *y* is either an array of intensity values for each of the 49 ROIs or a vector of source intensity values for each gray matter voxel). The inverse of the ICA-derived mixing matrix provides a set of loading values (*A*) for each subject *s* and each of the *M* components (*A*_*m*,s_; *m* = 1,2,3; s = 1,…,23). An approximation ($\widetilde {V_{{{\mathrm{T}}}}}$) of the original *V*_T_ data can be reconstructed as the sum of the products of component spatial intensity values and subject loading values ($y_mA_{m,s}$) and the subject’s global mean value ($\overline {V_{{{{\mathrm{T}}}},{{{\mathrm{s}}}}}}$):

$$\widetilde {V_{{{{\mathrm{T}}}},{{{\mathrm{s}}}}}} = y_1A_{1,s} + y_2A_{2,s} + y_3A_{3,s} + \overline {V_{{{{\mathrm{T}}}},{{{\mathrm{s}}}}}}$$

The product of loading value and spatial intensity of a given component provides a measure of *V*_T_-scaled source intensity, representing the contribution of that component to each subject’s overall KOR signal in each ROI in units of mL/cm^3^. Subject loading values are therefore proportional to but not a direct measure of the magnitude of KOR binding availability in regions represented in a given component, and will be referred to as indicating KOR signal. Regions where the product of a given ROI spatial intensity value and subject loading value for that source is positive indicate higher KOR signal (relative to subject mean), while negative values represent lower KOR signal.

ICA was performed using the Infomax algorithm [30], in line with previous protein PET studies [23–25]. Analyses were performed in R using the *‘ica’* package [31] for ROI-level analyses and in MATLAB using the structural brain mapping module of the GroupICA of fMRI toolbox (http://trendscenter.orgsoftware/gift/) [32] for voxel-level analyses.

### Stability of the ICA solutions

To assess the stability of ICA components, ROI-level ICA was repeated extracting different numbers of components (i.e., different model orders), as in prior work on ICA applied to PET data [25]. ICA was repeated extracting 2, 4, 6, or 9 components and source maps for the extracted components were compared with those from the primary analysis by assessing correlations in spatial intensity values across ROIs. To assess the stability of component extraction in voxel-level analyses, the Icasso approach was employed as implemented in the GroupICA toolbox [33]. Icasso provides measures of the statistical reliability of estimated components by iterating the extraction multiple times with different initial conditions. Twenty iterations were performed and repeatability of components was assessed using the cluster-quality index (*I*_q_), a summary metric of between-iteration similarity for each component ranging from 0 to 1 (representing identical solutions in every iteration) [33]. Previous work has used *I*_q_ > 0.8 as a threshold for reliably identified components in PET [25] and fMRI [34] data.

### Statistical analysis

Subject loading values for each component from ROI-level analyses were compared between HC and MDD groups using two-sided *t* tests or the Mann–Whitney *U* test in the case of non-normally distributed variables. Within the MDD group only, relationships between component loading value and symptom severity as measured by the HDRS and BDI were assessed using Pearson’s r or Spearman’s rho. Tests (group comparison and correlation with HDRS and BDI scores for loading values of each of three components) were considered significant at a Bonferroni-adjusted threshold of *p* < 0.0056 (0.05/9). In follow-up analyses, the same comparisons were performed with component loading values from voxel-level ICA. Concordance between ROI- and voxel-level analyses were assessed by computing correlations between source intensities by extracting mean spatial intensity from the same ROIs applied to voxel-level source maps and between subject loading values of each component.

## Results

### KOR distribution

Detailed participant and scan characteristics were published previously [14] and are summarized in the Supplementary Table. [^11^C]GR103545 *V*_T_ is high throughout limbic brain regions including anterior cingulate cortex, insula, hippocampus, and amygdala (Supplementary Fig. 1).

### ROI-level analyses

**Fig. 1:**
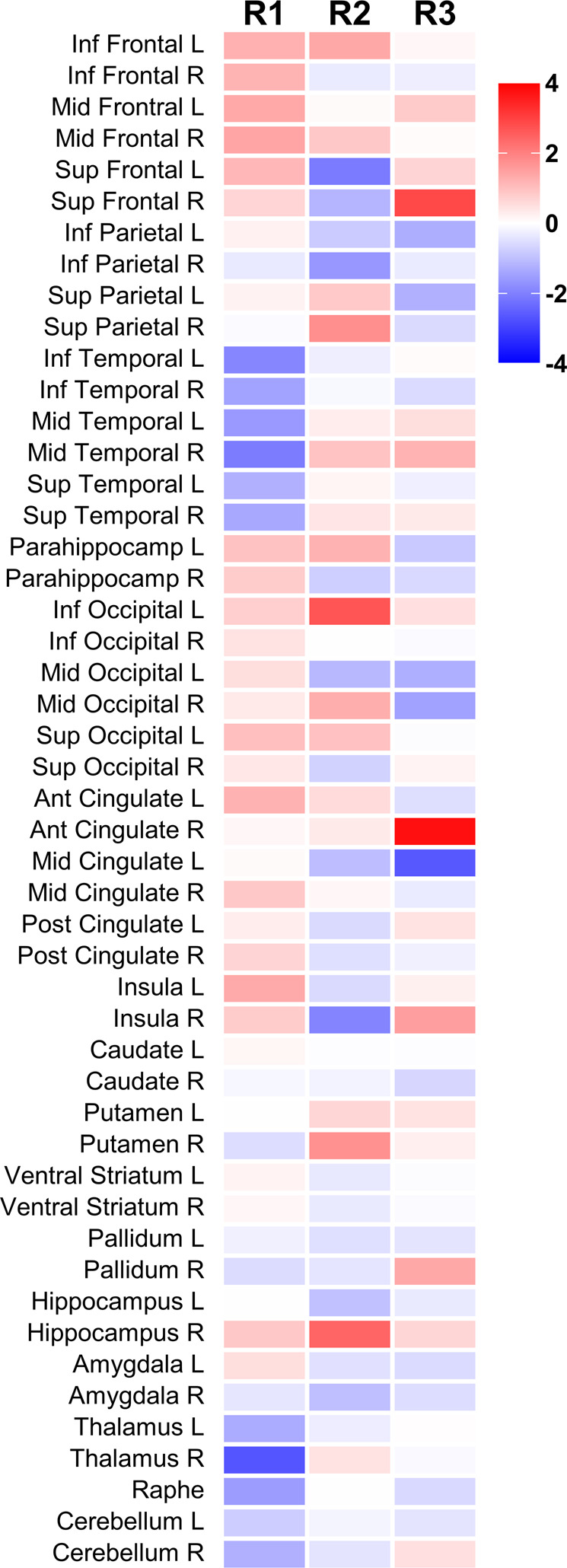
Regional patterns of source variance in [^11^C]GR103545 *V*_*T*_ from ROI-level ICA. Color bar is spatial intensity in arbitrary units.

**Fig. 2:**
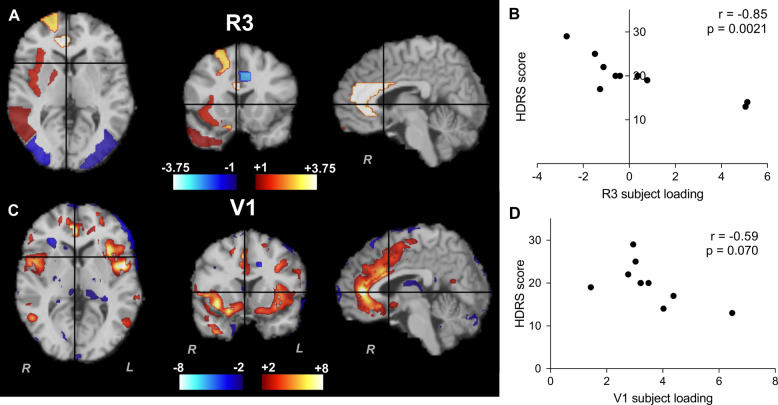
Sources of variance in KOR availability associated with depressive symptom severity. **A** Spatial pattern of ROI-level component 3 (R3). **B** Relationship between HDRS score and R3 subject loading in MDD subjects. **C** Spatial pattern of voxel-level component 1 (V1). **D** Relationship between HDRS score and V1 subject loading in MDD subjects. Color bars are spatial intensity in arbitrary units.

Three independent components of [^11^C]GR103545 *V*_T_ were extracted (Fig. 1), presented in descending order of the amount of variance in *V*_T_ each component accounts for across subjects. The first, R1, which accounted for 38% of the variance in V_T_ signal, was characterized by KOR availability bilaterally in frontal lobes, and with negative signal (relative to mean) in temporal lobes and thalamus. The second (R2; 11% of variance) had highest intensity in inferior occipital lobe along with right hippocampus, superior parietal lobe, and putamen. The third (R3; 9.2% of variance) was highly lateralized to the right side, with high positive signal in right anterior cingulate as well as right superior frontal gyrus and insula, and large negative values in left middle cingulate (Fig. 2A). Components with highly similar spatial source patterns were extracted when the analysis was repeated with model orders of 2, 4, 6, and 9 (Supplementary Fig. 2), indicating that these were reliably identified within this dataset. Component loading values were strongly correlated with [^11^C]GR103545 *V*_T_ in the ROI with highest spatial intensity value for that component (*p* < 0.0002; Supplementary Fig. 3), demonstrating the relationship between subject loading values and regional *V*_T_.

#### Effect of diagnosis

**Fig. 3:**
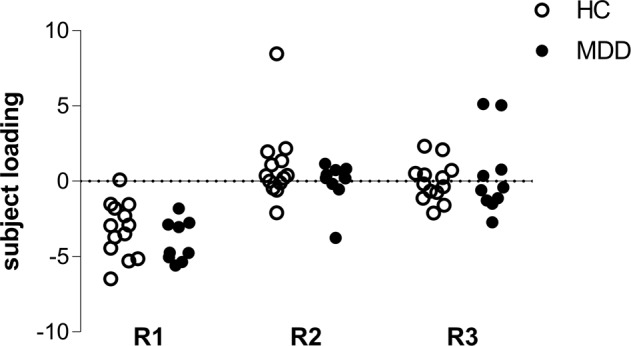
Subject loadings of components from ROI-level ICA in HC (open circles) and MDD (closed circles) subjects. No differences in loading of any ROI-level [^11^C]GR103545 *V*_T_ component was observed as a function of diagnosis (ps > 0.19).

There was not a statistically significant difference between MDD patients and HCs in mean whole-brain *V*_T_ (HC 15.8 ± 5.2 mL/cm^3^; MDD 18.9 ± 4.5 mL/cm^3^, *t* = 1.56, *p* = 0.13) or in subject loading values for any ICA component (Fig. 3; R1, *t* = 1.34, *p* = 0.19; R2, *U* = 54, *p* = 0.52; R3, *t* = 0.44, *p* = 0.67).

#### Effect of depression severity

Within the MDD group, R3 loading was negatively correlated with HDRS score (*r* = −0.85, *p* = 0.0021, Fig. 2B and Table 1). Thus, lower loading of this component – corresponding to lower KOR signal in right anterior cingulate, superior frontal cortex, and insula, and higher KOR signal in left middle cingulate – was associated with greater depression severity within the MDD group. This was confirmed by comparing HDRS scores with [^11^C]GR103545 *V*_T_ values in cingulate subregions directly (Supplementary Fig. 4). No statistically significant relationship was observed between R3 loading and score on the BDI (*r* = −0.28, *p* = 0.44) or between symptom severity scores and loading for any other component (ps > 0.41; Table 1).

**Table 1:** Correlations between symptom severity and subject loading in the MDD group.

HDRS	BDI	R1	R2	R3	V1	V2	V3	
1	0.37	−0.29	−0.10	−0.85**	**−0.57**^**†**^	**0.59**^**†**^	0.26	**HDRS**
	1	−0.20	0.20	−0.28	−0.28	−0.11	0.53	**BDI**
		1	0.091	0.059	0.078	−0.55	−0.75*	**R1**
			1	0.055	0.12	0.51	0.067	**R2**
				1	**0.57**^**†**^	**−0.60**^**†**^	0.21	**R3**
					1	−0.34	−0.44	**V1**
						1	0.15	**V2**
							1	**V3**

Bold values represent at least one of the thresholds of statistical significance.

Values are Spearman’s rho for pairs including R2 or V3 and Pearson’s *r* otherwise.

*HDRS* Hamilton Depression Rating Scale, *BDI* Beck Depression Inventory.

**p < 0.01; *p < 0.05; ^†^*p* < 0.1.

### Voxel-level analyses

**Fig. 4:**
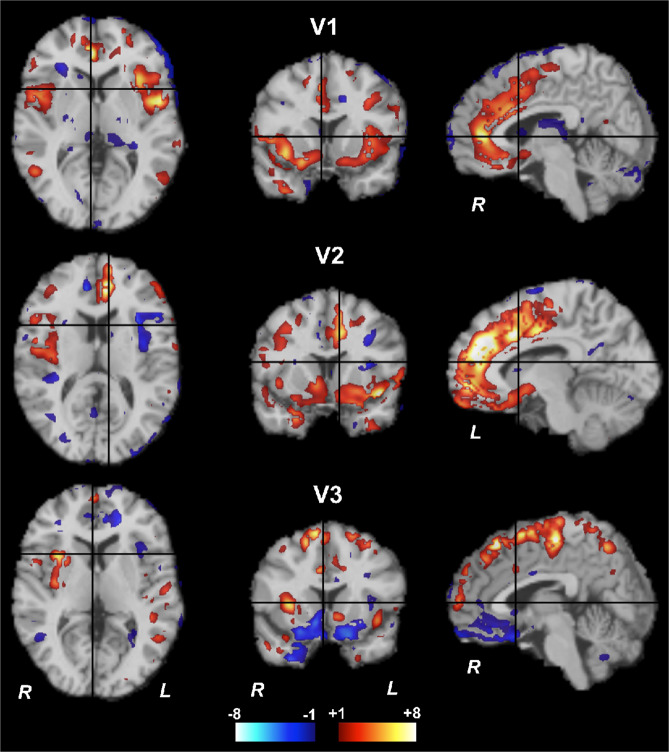
Sources of variance in [^11^C]GR103545 *V*_*T*_ from voxel-level ICA. Color bar is spatial intensity in arbitrary units. Each row of this figure depicts component loading values for one of three components identified from voxel-level ICA analysis.

ICA was repeated using voxel-wise images of [^11^C]GR103545 *V*_T_ to explore voxel-level patterns of variance. Again, three components were extracted (Fig. 4). Stability analyses found that each IC had *I*_q_ > 0.95, indicating that they are reliably extracted from the data. The component accounting for the largest amount of variance, V1, included right anterior cingulate cortex and ventral striatum as well as bilateral insula. V1 resembled R3 spatially (Fig. 2A, C). To confirm this similarity, spatial intensity values for each of the 49 ROIs used in the analyses above were extracted from the V1 source map and compared to R3 intensity values. These were correlated at a trend level (*r* = 0.28, *p* = 0.052), indicating that similar regional patterns were extracted despite the different level and patterns of variance in the voxel-level data. Accordingly, loading across subjects in the MDD group was also similar between V1 and R3 (*r* = 0.57, *p* = 0.083; Table 1). The second component, V2, was characterized by KOR signal in left cingulate cortex and insula and bilateral ventral striatum. V2 subject loading showed a trend-level negative relationship with R3 loading (*r* = −0.60, *p* = 0.069). V3 including signal in superior frontal cortex with negative signal in ventromedial prefrontal cortex. V3 subject loading was negatively correlated with that of R1 (rho = −0.75, *p* = 0.017).

#### MDD effects

As in ROI-level analyses, there were no significant differences in subject loading values between MDD and HC groups for any component (ps > 0.17).

#### Depression severity effects

We observed no significant relationships between loading of voxel-based components and depression severity measures. Trend-level relationships to depressive symptoms were observed, with V1 loading tending to be negatively correlated with HDRS score (*r* = −0.59, *p* = 0.070; Fig. 2D and Table 1) and V2 loading tending to be positively correlated with HDRS score (*r* = 0.59, *p* = 0.074).

## Discussion

In this study, we identified anatomically discrete components of correlated KOR signal quantified by PET imaging with [^11^C]GR103545. While subject loading of these ICA components did not differ as a function of diagnosis, loading of a component characterized by positive KOR signal in right anterior cingulate, right superior frontal gyrus, and right insula was inversely correlated with depression severity within the MDD group. This correlation between KOR signal and depression severity was in our hypothesized direction, and was not observed in our original analysis that was confined to only four small a priori ROIs (amygdala, raphe nuclei, hippocampus, and ventral striatum) [14]. Lower loading of this KOR component in more severely depressed patients may reflect increased dynorphin tone resulting in KOR downregulation or binding site occupancy in these regions, or alternatively may reflect primary effects of genetically/epigenetically driven variation in KOR expression. While these data require replication in a larger independent sample, they speak to the power of ICA-based approaches to reduce the dimensionality of PET data and increase statistical power to identify regional patterns with relevant clinical correlates.

### Stability and Interpretation of ICA-derived Components of KOR Binding

Independent components of [^11^C]GR103545 *V*_T_ may reflect true biological units of differential KOR/dynorphin regulation, given the regionally specific release and regulation of these peptides. Indeed, post-mortem work suggests regionally specific effects of childhood abuse on KOR binding, with low binding in those with a history of childhood abuse observed in anterior insula but not thalamus or anterior cingulate cortex [35]. This appeared to be epigenetically driven, with decreased methylation at the 2^nd^ intron of *KOR* in those with a history of childhood abuse. While these previous data are partially consistent (anterior insula) and partially in conflict (anterior cingulate) with the current depression severity findings, they validate the notion of regionally specific regulation of KOR that may be epigenetically or genetically driven. However, the component structure of the present analyses should be considered preliminary until evaluated in an independent dataset, which to our knowledge is not yet available. Reliability of these findings *within* the present data was assessed in several ways. For ROI-level analyses, R1 and R3 were repeatable across different model orders when at least 3 components were extracted (Supplementary Fig. 2). In voxel-level analyses, stability analyses indicated that each of the 3 components were reliably extracted across 20 iterations of the analysis. Thus, the component structures presented here seem to be stable representations of these data. Further, comparison of ROI- and voxel-level analyses suggest consistent findings. The spatial map of V1 and V2 included regions represented in component R3 (notably including the right anterior cingulate in V1 and left anterior/middle cingulate in V2), and had trend-level associations with symptom severity that were directionally consistent with those seen with R3 (a negative trend with V1 loading and a positive trend with V2). This suggests that similar regional patterns were identified in voxel-level analyses, but due to the different variance patterns and weighting of the data these were spread across separate components. ROI- and voxel-level analyses also show different correlation levels between subject loadings within the same analyses (Table 1). This may reflect the vastly greater number of data points in voxel-wise analyses, which may have been less susceptible to single extreme points but less successful at separating fully independent components within this modest sample. Given these differences in data structure, concordance in the regions identified and the direction of their relationship with HDRS scores increases our confidence that these analyses are reliably identifying important sites of KOR variation. Therefore, while future work to validate these component structures may be informative about the organization of KOR/dynorphin signaling, and we speculate further on their biological relevance below, the primary outcome of these analyses is robust, data-driven identification of regions relevant to depressive symptoms within this sample.

### Anatomic distribution of depression severity finding

The component/group of ROIs, R3, that was inversely correlated with depression severity quantified by the HDRS included strong weightings from right anterior cingulate, right superior frontal gyrus (including dorsomedial prefrontal cortex), and right insula. Each of these regions has been previously associated with the pathophysiology of depression from neuroimaging studies. Specifically, a recent meta-analysis of resting-state fMRI (rsfMRI) found increased amplitude of low frequency fluctuations signal in MDD patients in an overlapping set of regions as those positively weighted in this KOR component, including right superior frontal gyrus, anterior cingulate, and insula, although the insula finding was bilateral [36], suggesting higher basal activity in these regions. In terms of functional effects of KOR in these regions, animal work has demonstrated that activation of KOR in medial prefrontal cortex inhibits local dopamine release, consistent with the role of KOR in ventral striatum [37].

R3 contained some lateralized weightings. This is not likely to be accounted for by handedness of the participants, as there was no evidence of a difference in R3 loading between right- and left-handed individuals (right-handed [*n* = 16, see Supplementary Table], mean −0.50 ± 1.89; left-handed [*n* = 6], mean −0.76 ± 1.71). In some cases, the weighting was in the same direction but stronger on the right than on the left (e.g., superior frontal gyrus); in others, the finding was in opposing directions on right vs. left sides (e.g., anterior cingulate, also reflected in V1 and V2). Moreover, the left mid-cingulate was weighted in the opposite direction in the factor as the right anterior cingulate, i.e., KOR signal in left mid-cingulate was *positively* correlated with depression severity quantified by HDRS, suggesting possible functional differentiation of KOR effects not just by hemisphere but along the anterior-posterior axis of the cingulate. Rostral anterior cingulate has been associated with treatment outcome with a broad range of antidepressant treatment interventions [38]. Mid-cingulate has been associated with monitoring outcomes of social decision-making [39], cognitive control, processing of negative affect, and of pain [40, 41]. Anterior cingulate cortex is related to a broad range of functions, including pain processing [42]. Specifically, the right anterior cingulate has been associated with the subjective experience of both physical and emotional pain [43, 44], and the distressing awareness of sympathetic activation and arousal [45]. Known findings implicating KOR distribution in cingulate to psychopathology in vivo and *post mortem* are reviewed below.

The observations of lateralized findings within R3 (positive weighting for right anterior cingulate, negative weighting for left mid cingulate) are not without precedent. It has been theorized that hemispheric asymmetry, especially in anterior cingulate, insula, and frontal lobe regions, is a shared feature among psychiatric disorders such as depression that are characterized by emotion dysregulation, poor motivation, and reactive coping styles [43, 45, 46]. Lateralized distribution of the serotonin 1 A receptor has been observed in frontal regions via PET imaging [47]. In the opioid system, a recent PET study found substantial lateralization of mu opioid receptor availability across the brain in healthy people [48] and a post-mortem study found marked differences between hemispheres in the concentration of several opioid peptides (but not in levels of mRNA encoding opioid receptors) in the anterior cingulate [49]. The present results, while preliminary, suggest that such hemispheric differences in opioid signaling may be clinically important.

Each of these components R3, V1, and V2, which were correlated with depression severity (V1 and V2 at trend level), substantially overlap with the functional salience network (SN; see Supplementary Fig. 5), including the anterior insula and dorsal anterior cingulate cortex [50]. The SN plays a critical role in conflict monitoring and orienting attention toward behaviorally salient stimuli [50] by regulating the within- and between-network activity of the central executive network (CEN) and the default mode network (DMN) [51, 52]. Aberrations of the SN appear to be a common feature across a wide range of psychiatric disorders, including depression [53] and the SN has been proposed as a clinical target for neuromodulatory treatments such as transcranial magnetic stimulation [54], especially in patients with high baseline SN connectivity with the dorsolateral prefrontal cortex [55]. The reported direction of SN differences in depression has been mixed (for example, both hyper- [56] and hypo- [57, 58] connectivity have been reported), possibly as a result of heterogeneity in depression. For example, SN connectivity with both DMN and CEN can distinguish between patients with depression and a history of suicide attempt and those without [59, 60]. The SN has been linked with symptoms such as disrupted cognitive-attentional processing, reduced reward sensitivity, and dysphoria [54]. Component R3 also included the superior frontal gyrus, an area which shows heightened connectivity with SN in both depression-affected and non-affected adolescents with a family history of depression [61]. Translational work on the KOR system in psychopathology in depression-related behavior has focused on modulation of reward circuitry via dopaminergic outputs from ventral tegmental area to nucleus accumbens;[3] the current finding supports further investigation of the role of KOR across a broader set of regions and networks in the pathophysiology of depression in humans.

### Comparison to existing KOR literature

There is a dearth of in vivo human study quantifying KOR in depression. Interestingly, a recent study of patients with alcohol use disorder (AUD) found that KOR availability assessed via PET imaging with the antagonist radiotracer [^11^C]LY2795050 found some convergent findings in terms of anatomic localization and direction of effect with the current report: baseline KOR availability in *cingulate cortex* and *insula* (as well as regions not identified in the current study, including hippocampus, amygdala, and striatum) was *inversely* associated subsequent clinical improvement (reduction in craving) following treatment with the nonspecific opioid antagonist naltrexone [21]. One possible interpretation of those findings is that excessive dynorphin signaling in these regions in AUD patients led to KOR downregulation observed by PET, and that this excessive dynorphin signaling was effectively blocked by subsequent naltrexone treatment in these regions. AUD is highly comorbid with MDD, such that approximately 30–40% of individuals with either diagnosis will also experience the other during their lifetime [62]. To the extent that lower KOR availability in anterior cingulate and insula is implicated in both AUD and in depression severity, it may suggest dynorphin signaling as a common pathway impacting both conditions, potentially through mediating stress responses [63].

A recent postmortem study found that, in comparison to tissue from healthy individuals who died from accidental causes, individuals with a history of severe childhood abuse who died by suicide had lower KOR expression in anterior insula specifically, partially consistent with the anatomic localization of the current finding and in the same direction; this effect was not observed in anterior cingulate or mediodorsal thalamus [35]. Other post-mortem findings have identified *elevated* prodynorphin mRNA in the caudate nucleus of suicide victims as compared to healthy volunteers; [64] *lower* prodynorphin mRNA levels in the amygdala among subjects with MDD and bipolar disorder compared to controls; [65] and *no difference* in prodynorphin or KOR mRNA between patients with major depressive disorder and healthy volunteers [66]. These postmortem studies had limited ability to conduct psychiatric state-based phenotyping to relate KOR or prodynorphin levels to depression severity.

### Strengths and limitations

As discussed above, few studies to date have studied the regional role of KORs in human mood disorders, motivating us to explore the possible contribution of KOR signaling outside the subcortical structures assessed originally. Several features of these data made ICA an advantageous approach for exploratory secondary analyses. Along with the small sample size, variability in quantitative measures of KOR binding with the [^11^C]GR103545 radiotracer present a challenge in small, high-binding regions and in conventional voxel-level analyses [18]. ICA was therefore well-suited as a data-driven approach to identify important, coherent sources of variance less susceptible to noise in any one region. Reducing whole-brain *V*_T_ data to maximally independent components allowed us to both identify important regional relationships, and to capture cross-regional patterns that provide a preliminary indication of how KOR function may be organized across the brain. These analyses highlighted the importance of KOR/dynorphin in regions that, while not hypothesized a priori, are known to play a key role in mood regulation. It is notable that the initial hypotheses were based largely on evidence from rodent models, which may provide a weaker analog of cortical function compared to highly conserved subcortical regions.

Limitations to this analysis include the small sample size and limitations of the [^11^C]GR103545 radiotracer, which has slow kinetics contributing to variability in parameter estimates. Future work on this system can take advantage of radioligands with improved kinetic properties [67, 68]. Given the small sample size and the fact that these data represent the only PET study to date of KOR binding in MDD, we were unable to confirm the reproducibility of this component structure through cross-validation within this sample or in an independent one. We thus interpret these spatial patterns of KOR binding availability as preliminary. We applied ICA methods that have previously been evaluated in PET data, but other algorithms and variations are available such as adaptive mixture ICA [69] which may have provided different and potentially more robust solutions. Finally, it is not possible in these data to distinguish between differences in receptor density and differences in endogenous neurotransmitter concentration, both of which can affect in vivo binding availability.

## Conclusions

This study represents the most direct data to date relating quantification of the kappa opioid system in vivo to depression severity. These data highlight regions and networks of KOR/dynorphin binding that merit further study in larger samples. If replicated, these results may suggest that the kappa opioid system can contribute to more severe forms of depression or reflect the consequences of stress due to more severe depression. These networks may be particularly relevant to the somatic and behavioral symptoms of depression captured preferentially by the HDRS. Furthermore, KOR quantification via PET may identify depressed patients with a kappa opioid abnormality, who may be more likely to benefit from kappa-modulating interventions.

## Supplementary information

Supplemental material
