# Supplementary material for: Data-driven analysis of kappa opioid receptor binding in major depressive disorder measured by positron emission tomography
Source: Transl Psychiatry. 2021 Nov 27;11:602. doi: 10.1038/s41398-021-01729-5 (PMC8627509; doi:10.1038/s41398-021-01729-5)
Supplement: Supplementary file 1 — Supplemental material [file 41398_2021_1729_MOESM1_ESM.pdf]

<u>**Supplemental Material**</u>

Data-driven Analysis of Kappa Opioid Receptor Binding in Major Depressive Disorder Measured by Positron Emission Tomography

Smart K, Yttredahl A, Oquendo MA, Mann JJ, Hillmer AT, Carson RE, Miller JM

Contents:

Supplemental Table. Participant and scan characteristics.

Supplemental Figure 1. KOR availability across the brain measured by [^{11}C]GR103545 VT, median of 23 subjects.

Supplemental Figure 2. Regional patterns of source variance in [^{11}C]GR103545 V_T from ROI-level ICA using different model orders.

Supplemental Figure 3. Subject loading values for each component reflect V_T within the contributing ROIs.

Supplemental Figure 4. ROI V_T values in cingulate cortex subregions represented in R3 vs. HDRS scores in the MDD group.

Supplemental Figure 5. Voxels included in component V1 overlapping with the salience network.

Supplemental References

Supplemental Table. Participant and scan characteristics.

	MDD	HC	*p*
N (n female)	10 (5)	13 (6)	>0.99
Age, years	32.6 ± 6.5	34.8 ± 10.0	0.54
Handedness	7 right, 2 left, 1 unknown	9 right, 4 left	
Depressive symptoms			
17-item Hamilton Depression Rating Scale (HDRS)	19.9 ± 4.8	0.67 ± 1.2	$< 10^{-9}$
Beck Depression Inventory (BDI)	29.3 ± 10.2	0.17 ± 0.58	$< 10^{-8}$
Scan characteristics			
Injected activity, MBq	419 ± 221	431 ± 151	0.88
Injected mass, ng/kg	8.18 ± 5.85	5.77 ± 3.52	0.22

Values are mean ± SD. P values are from Fisher's exact test (sex) or t tests comparing MDD and control groups.

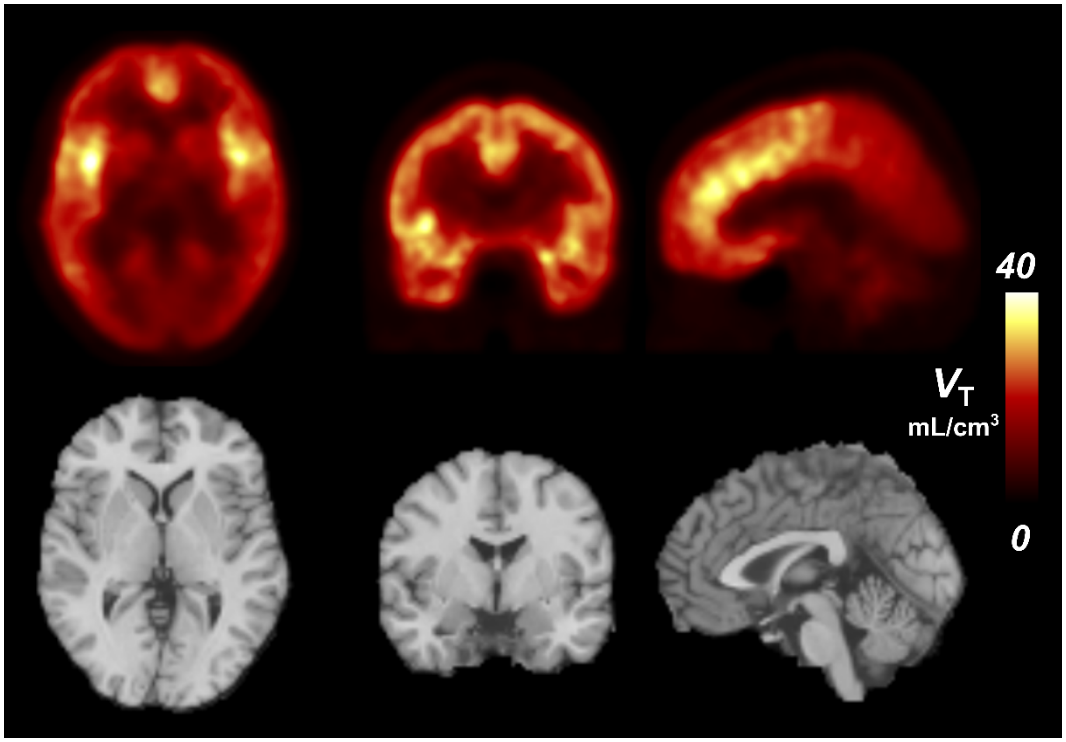

Supplemental Figure 1. KOR availability across the brain measured by [^{11}C]GR103545 V_T, median of 23 subjects.

ICA component stability

Source maps for ICA with model orders 2, 4, 6, and 9 are shown below. With model order 2, components are similar to R1 and R2 from the primary analysis (main text Fig. 1; *r*s > 0.98, *p*s < 10^{-8}). Components with source maps resembling main text R1 and R3 were reliably extracted with model orders 4, 6, and 9 (R1 and R4.1, r = 0.79; R3 and R4.3, r = 0.85; R1 and R6.1, r = 0.88; R3 and R6.4, r = 0.92; R1 and R9.1, r = -0.72; R3 and R9.5, r = 0.79; all p < 10^{-5}).

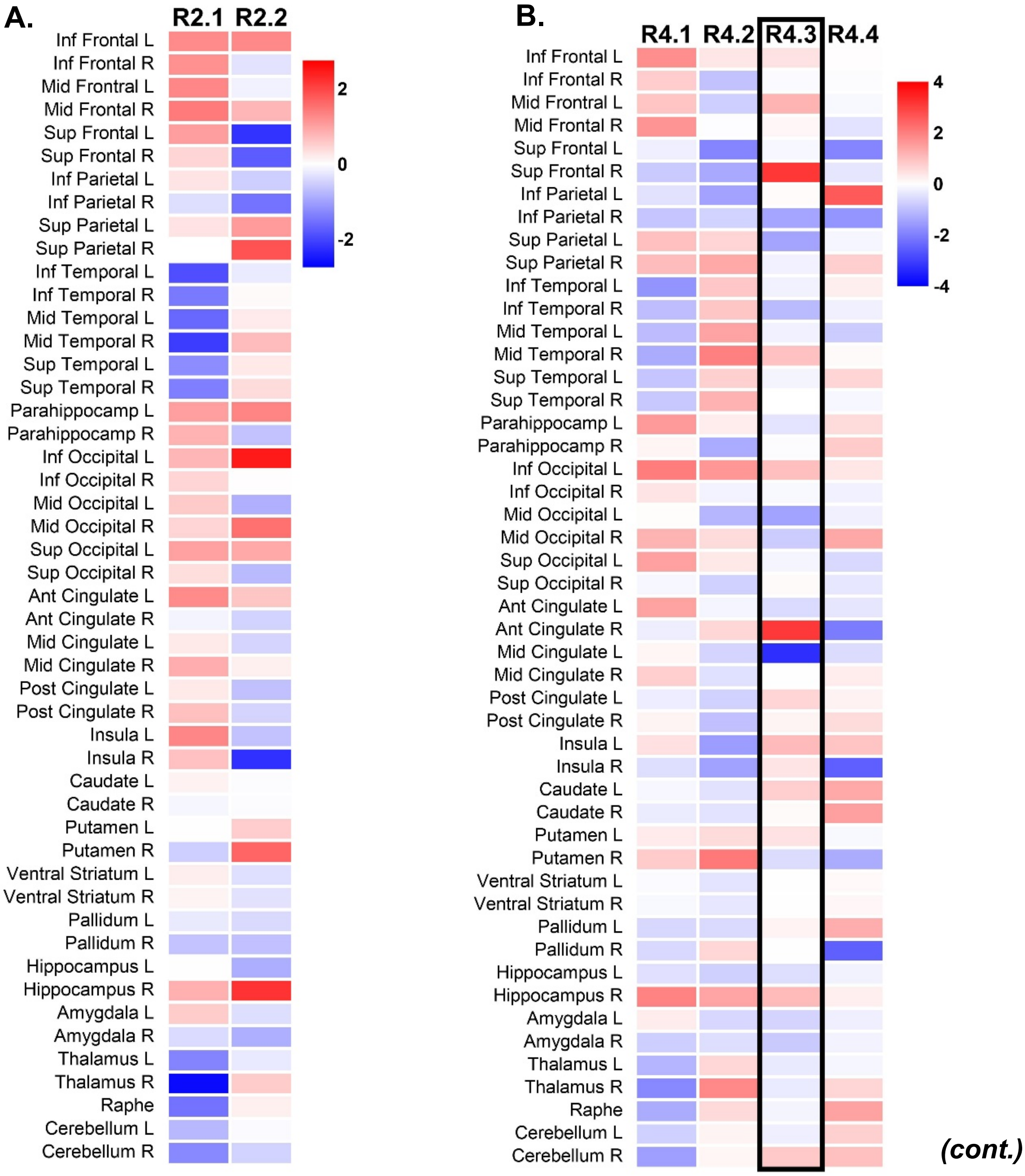

(cont.)

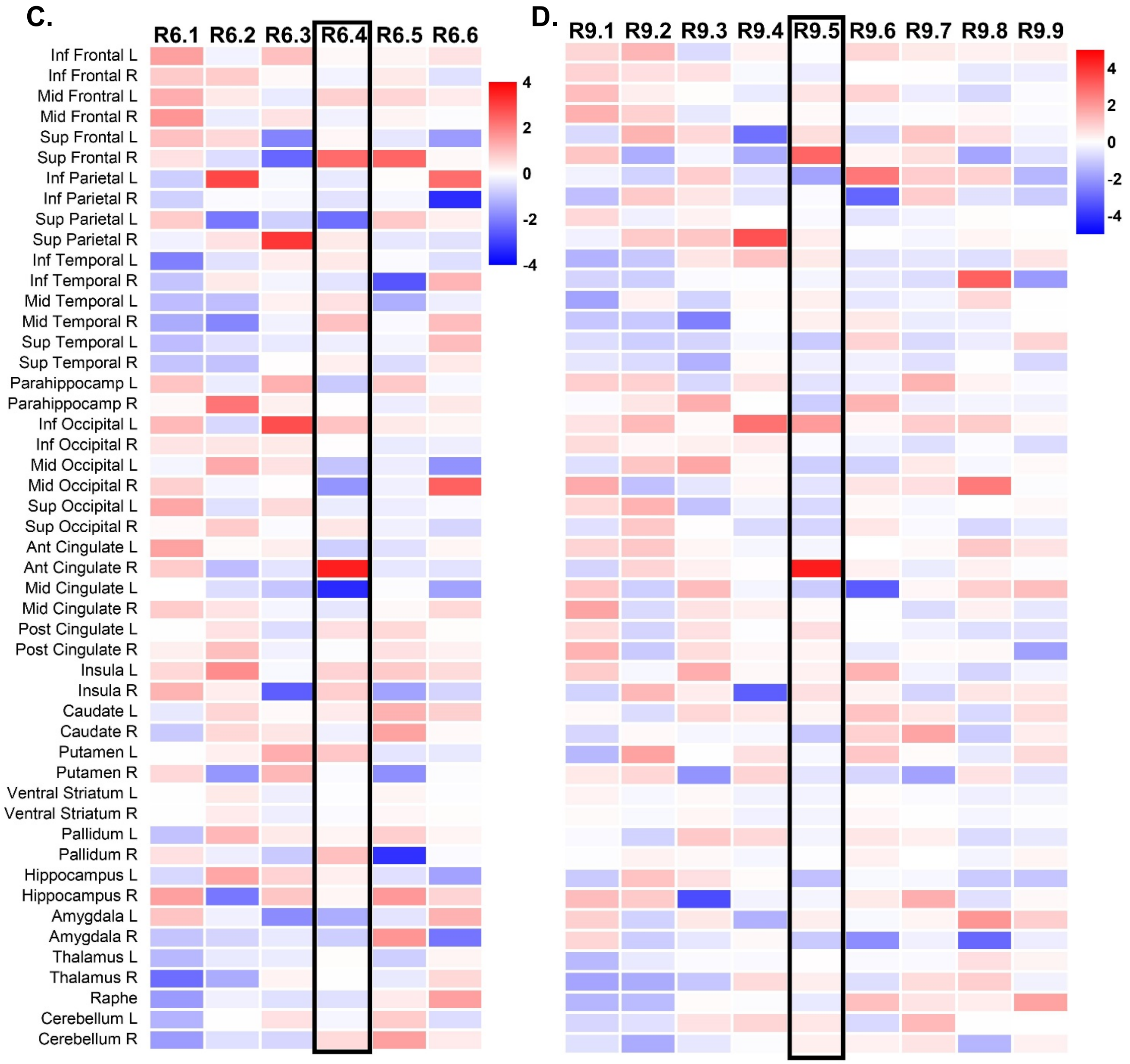

Supplemental Figure 2. Regional patterns of source variance in [^{11}C]GR103545 V_T from ROI-level ICA using different model orders. Color bar is spatial intensity in arbitrary units. With 2 components (panel A), source maps resemble R1 and R2 from the primary analysis (main text Fig. 1). A component driven by positive signal in right anterior cingulate and negative signal in left mid cingulate, similar to R3 in the main analysis, is seen with 4 (B), 6 (C), and 9 (D) components extracted (boxes).

<u>Subject loading correlation with ROI V_T</u>

Component loading values are proportional to signal intensity in regions represented in the component. To illustrate this, correlations were computed between the loading value of each component and V_T in the ROI with the greatest absolute source intensity within that component: for R1, right thalamus, for R2, left inferior occipital lobe, and for R3, right anterior cingulate cortex (main text Fig. 1).

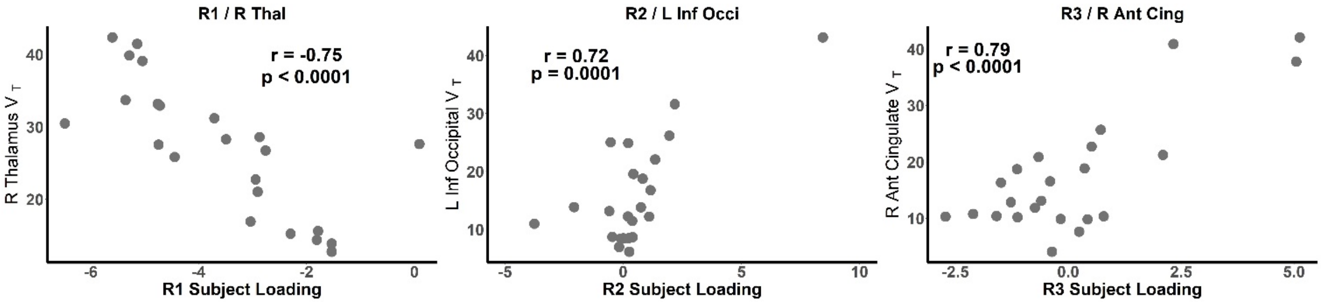

Supplemental Figure 3. Subject loading values for each component reflect V_T within contributing ROIs. Subject loading values for components R1-R3 are plotted against V_T value in the ROI with the highest absolute source intensity within that component (main text Figure 1): for R1, right thalamus (R1 spatial intensity -2.67), for R2, left inferior occipital lobe (R2 spatial intensity +2.66) and for R3, right anterior cingulate cortex (R3 spatial intensity +3.76). This illustrates that subjects with higher R1 loading values have *lower* V_T relative to the rest of the group in regions with negative spatial intensity, particularly the right thalamus, while subjects with higher R2 and R3 loading values *higher* V_T in ROIs with positive spatial intensity.

ROI V_T correlation with HDRS scores

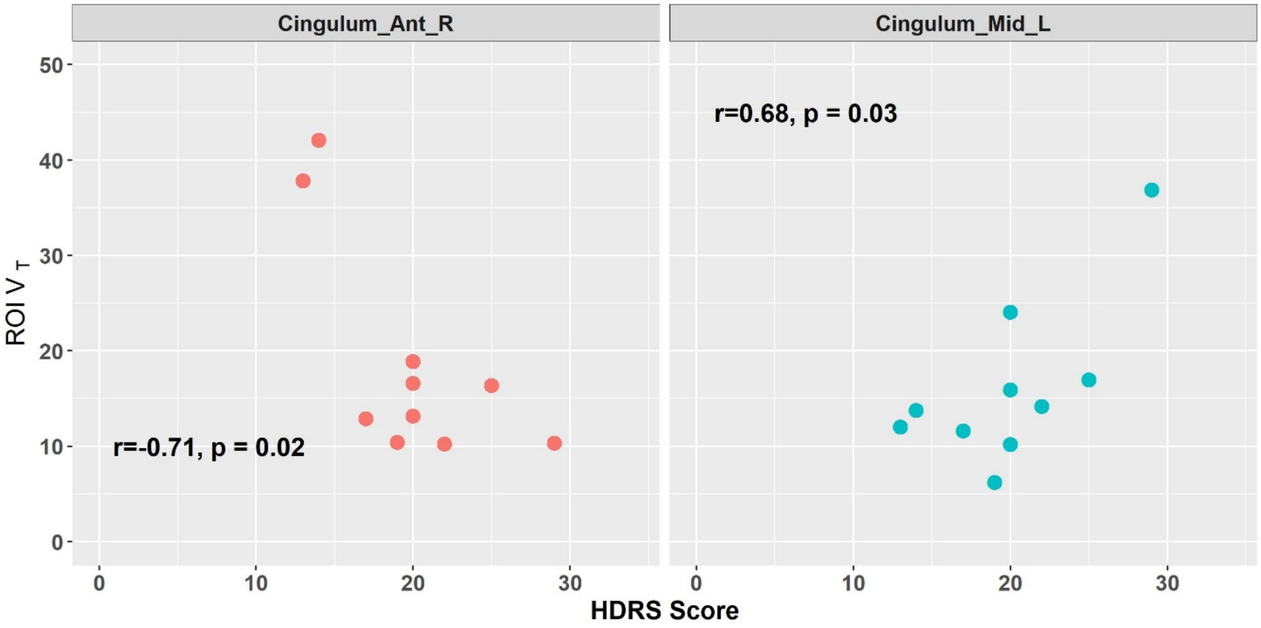

Supplemental Figure 4. ROI V_T values in cingulate cortex subregions represented in R3 vs. HDRS scores in the MDD group. Right anterior cingulate and left middle cingulate cortex show the expected opposing relationships with HDRS scores across subjects.

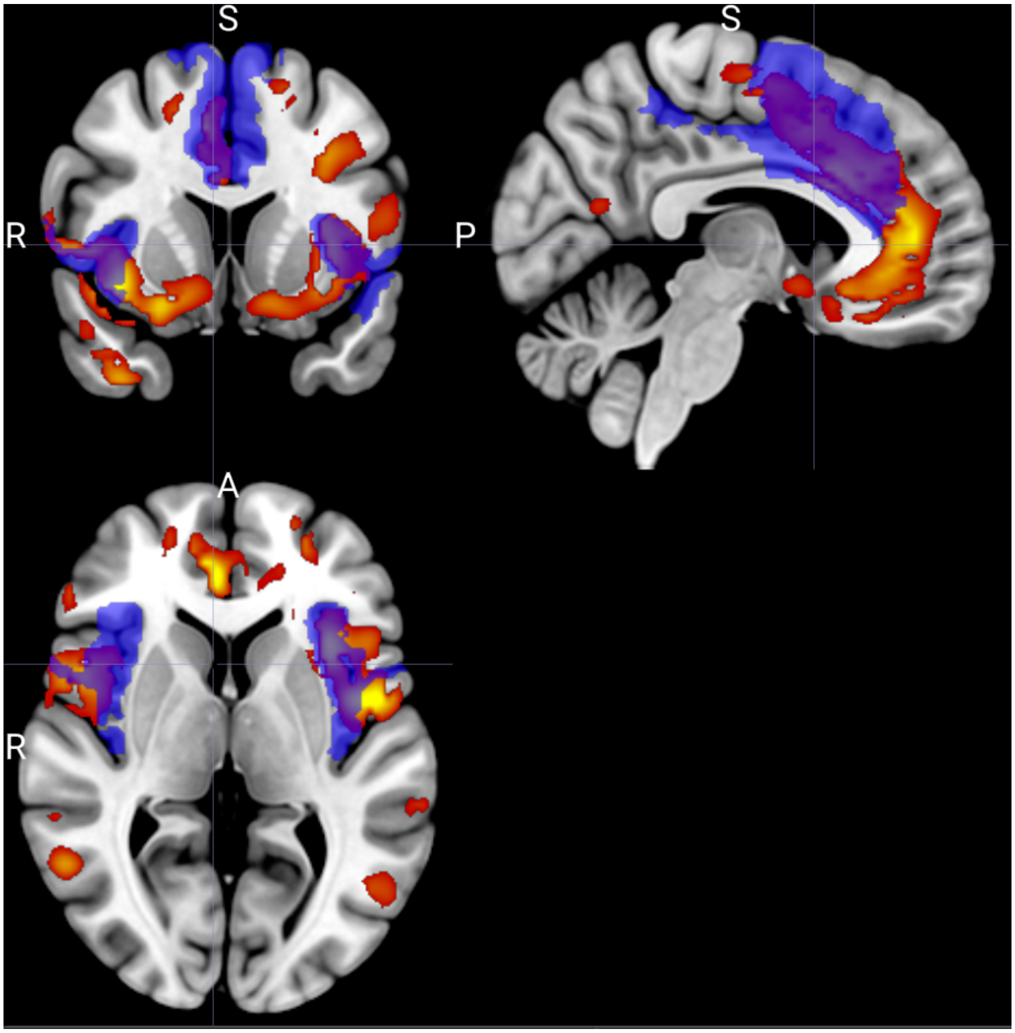

Supplemental Figure 5. Voxels included in component V1 overlap substantially with those included in the salience network. Twelve parcels from the 7-network 100-parcel Schaefer parcellation atlas [1] corresponding to the salience network [2] were combined into a single volume of interest, shown in blue. Spatial pattern for component V1 shown in yellow/red.

Supplemental References

1. Schaefer, A., et al., *Local-Global Parcellation of the Human Cerebral Cortex from Intrinsic Functional Connectivity MRI.* Cerebral Cortex (New York, NY), 2018. **28**(9): p. 3095-3114.
2. Yeo, B.T.T., et al., *The organization of the human cerebral cortex estimated by intrinsic functional connectivity.* Journal of Neurophysiology, 2011. **106**(3): p. 1125-1165.
